# Bi-criteria Acceleration Level Obstacle Avoidance of Redundant Manipulator

**DOI:** 10.3389/fnbot.2020.00054

**Published:** 2020-10-15

**Authors:** Weifeng Zhao, Xiaoxiao Li, Xin Chen, Xin Su, Guanrong Tang

**Affiliations:** ^1^Foshan Longshen Robotics LTD., Foshan, China; ^2^Guangdong Key Laboratory of Modern Control Technology, Guangdong Institute of Intelligent Manufacturing, Guangzhou, China

**Keywords:** recurrent neural network, path planning, redundant manipulator, acceleration level obstacle avoidance, bi-criteria

## Abstract

In this paper, an improved obstacle-avoidance-scheme-based kinematic control problem in acceleration level for a redundant robot manipulator is investigated. Specifically, the manipulator and obstacle are abstracted as mathematical geometries, based on the vector relationship between geometric elements, and the Cartesian coordinate of the nearest point to an obstacle on a manipulator can be found. The distance between the manipulator and an obstacle is described as the point-to-point distance, and the collision avoidance strategy is formulated as an inequality. To avoid the joint drift phenomenon of the manipulator, bi-criteria performance indices integrating joint-acceleration-norm minimization and repetitive motion planning is adopted by assigning a weighing factor. From the perspective of optimization, therefore, an acceleration level quadratic programming (QP) problem is eventually formulated. Considering the physical structure of robot manipulators, inherent joint angle, speed, and acceleration limits are also incorporated. To solve the resultant QP minimization problem, a recurrent neural network based neural dynamic solver is proposed. Then, simulation experiments performing on a four-link planar manipulator validate the feasibility and effectiveness of the proposed scheme.

## 1. Introduction

With the advances of society, ranging from industry to military, home furnishing, service, medical treatment, *etc*., robot technology has already become gradually mature. Simultaneously, the high demand on the execution abilities of a robot manipulator working in complicated environment also poses a challenge to robotic control. Due to its degrees of freedom (DOF) exceeding ones required by the robot to complete the given tasks, a redundant manipulator shows better flexibility, multifunction, and wide universality than the traditional non-redundant robot.

As a fundamental problem in robotic control, the kinematic motion planning problem of the redundant manipulator has already been widely investigated in recent years. Series of related products have been reported, e.g., in Li et al. (2016), from the perspective of game theory, and a distributed recurrent neural network (RNN)-based dynamic controller was proposed for the coordination control of multi-robot system. In Li et al. (2020), based on the RNN, Li et al. investigated the kinematic control problem of the multi-robot system under neighbor-to-neighbor communication. To access the desired global command, an observer was developed for estimating the velocity information of the desired motion trajectory. A distributed RNN scheme was proposed in Jin et al. (2018) for the consensus and cooperative control of a multiple manipulator under limited communication, achieving the global cooperation of PUMA 560 manipulators. The kinematic control of a redundant manipulator disturbed by the periodic input was investigated in Zhang et al. (2019a) based on the RNN. Moreover, Zhang et al. proposed an RNN control scheme incorporating the joint acceleration constraint for the redundant manipulator in Zhang et al. (2019b), which is solved in acceleration level. In Xu et al. (2019a) and Xu et al. (2020), the RNN was used to the kinematic control of redundant manipulator with model uncertainties and coupling of motion and contact force, respectively. In Chen et al. (2019), the RNN was applied to the motion control of a mobile robot. In Li et al. (2018a) and Li et al. (2018b), a modified RNN-based controller was proposed for motion control of the manipulator disturbed by noises. In Chen et al. (2020a), a time-varying noise disturbance rejection constraint was established. In addition, Chen et al. proposed a joint velocity, acceleration, and joint jerk three-level simultaneous minimization scheme in Chen et al. (2020b). The abovementioned involve single and multiple robot systems. Following them, the RNN can in principle handle the kinematic problem of a redundant manipulator. In addition, in most of the abovementioned literature, the consensus is that the quadratic programming (QP) method, i.e., where the manipulator kinematic control problem is described as a QP minimization problem, is adopted, owing to which can incorporate physical constraints such as joint angle and joint velocity limits.

When performing a desired task, the success of the motion planning task may not be guaranteed if the manipulator encounters a sudden obstacle, and even the robot manipulator will be damaged due to the collision. The obstacle avoidance problem of a redundant manipulator is thus worthy of investigation. Obstacle avoidance, called collision avoidance, always plays an important role and is continuously investigated among redundant manipulators. For collision avoidance, in general, two aspects need to be considered: one is robot-to-environment, and the other is robot-to-robot. Especially for a multi-robots system, the obstacle avoidance scheme should include not only the collision avoidance between robot arms but also the collision avoidance between robots and environmental obstacles. Many obstacle avoidance methods have been proposed, such as pseudo-inverse-based ones (Zlajpah and Nemec, 2002; Lee and Buss, 2007; Guo et al., 2018), random-sampling-based methods such as rapidly exploring random tree (RRT) (Ju et al., 2014; Zhang et al., 2018), artificial potential field (Volpe and Khosla, 1990; Kim and Khosla, 1992), and QP-based optimization methods (Zhang and Wang, 2004; Guo and Zhang, 2014, 2019; Zhou et al., 2019; Xu et al., 2019b). In general, pseudo-inverse methods have no ability of handling physical structure constraints of a manipulator. The RRT methods are very effective for high-dimensional and complicated environments, which makes the generated path approaches a collision-free region by randomly sampling unknown space. For ones aided with an artificial potential field, different environments need specialized potential functions. Among such a method, the robot is assumed to move within a virtual force field where the target and the obstacle are denoted as an attractive pole and a repulsive surface, respectively. Although effective, these two methods are accompanied by higher computational costs; for the latter, the computational complexity is exponentially increasing to the DOFs of the robot.

Generally speaking, for QP-based methods, the obstacle avoidance strategy is usually formulated as an attachment constraint of the resultant QP minimization problem. For their works in Zhang and Wang (2004), Guo and Zhang (2014, 2019), etc., the collision avoidance constraints were set inner and outer thresholds for safety. In Xu et al. (2019b) and Zhou et al. (2019), a relatively simple inequality that can avoid collision with the obstacle was proposed. In their works, both the obstacle and manipulator are abstracted as point sets. A safe distance is given by ensuring the distance between the manipulator and obstacle is always greater than the safe distance, and the safety is ensured. However, as points representing the manipulator are chosen in a uniform way, this method carries a possible risk that the chosen points do not collide with the obstacle; in practice, the collision has already happened due to the distance from the chosen point to the obstacle may be greater than the shortest distance between the manipulator and the obstacle.

In this study, therefore, we provide an improved obstacle avoidance scheme that can determine the nearest point on every link of the manipulator to the obstacle. By always keeping the minimal distance between them outside the non-safety region, the safety is ensured. In addition, if the acceleration vector is quite different at the front and back time, it will produce excessive velocity, which will enable the manipulator to shake, critically impact, or even cause damage to the manipulator or potential safety accident. Moreover, if not taking the joint acceleration into account, the generated joint velocity command may be discontinuous (Guo and Zhang, 2014, 2019). Consequently, in this study, the kinematic control problem of a redundant manipulator is investigated in terms of acceleration level. Specifically, the robot manipulator and obstacle are first abstracted as mathematical geometries based on the vector relationship between geometric elements in the search for the Cartesian coordinates of the points whose distance from every link of the manipulator to the obstacle is shortest. The distance between a robot manipulator and an obstacle is described as point-to-point distance, and an inequality constraint is thus constructed, which is built in acceleration level, to avoid the obstacle. To avoid the joint drift problem and improve the stability and reliability of robots in periodic tasks such as palletizing, welding, etc., the bi-criteria performance indices integrating joint-acceleration-norm minimization (MAN) and repetitive motion planning (RMP) is considered by assigning a weighing factor. The kinematic control problem of the manipulator is transformed into an equality constraint mapping from Cartesian space to joint acceleration space. To sum up, an acceleration-level quadratic programming (QP) problem is obtained, combining the joint angle, joint velocity, and joint acceleration limits rebuilt in the acceleration level. Then, utilizing the real-time property of the RNN, we designed an RNN based neural dynamic controller to solve the QP problem. Finally, simulative experiments are performed on a four-link planar manipulator, validating the feasibility of the proposed control scheme and obstacle avoidance strategy by simulative results.

The ensuing part of this paper is arranged around the following aspects: preliminaries such as kinematic description of redundant manipulator, the nearest point selection as well as the formulation of the inequality obstacle avoidance strategy, and problem statement are introduced in section 2. Section 3 shows the QP problem reformulation and the design of RNN controller. Simulation results are given in section 4, where both the static and dynamic obstacle are considered. Section 5 summarizes the whole paper with a final remark. The main contributions of this paper are summarized as follows:

1. The acceleration-level kinematic control problem of redundant manipulator with the obstacle collision avoidance is investigated. Bi-critic performance indices consisting of joint-acceleration-norm minimization and repetitive motion planning are considered in order to avoid the joint drift and improve the stability and reliability of robots in periodic tasks.
2. An improved obstacle avoidance strategy that can return the nearest point of every link of a manipulator to the obstacle is proposed. By keeping minimal distance between the robot and the obstacle outside the non-safety region all the times, the safety is ensured.
3. An RNN-based dynamic controller combining the motion planner, obstacle avoidance and joint angles, joint speed, as well as joint acceleration constraints is proposed. Under its control, the robot achieves the desired trajectory tracking task with a desired tracking error, and it successfully avoids collision with static and dynamic obstacles.

## 2. Preliminaries and Problem Statement

### 2.1. Kinematics Description of Redundant Manipulator

For path planning task of a robot manipulator, the position of its end-effector is only determined by its joint space vector θ(*t*), and the relationship between them is usually described as

(1) $$\begin{array}{l} r\left(t\right)=f\left(\theta \left(t\right)\right), \end{array}$$

where *r*(*t*) ∈ ℝ^*m*^ are Cartesian coordinate of the end-effector at time *t*, and θ(*t*) ∈ ℝ^*n*^ are the coordinate of the end-effector in joint space. *f*(·): ℝ^*n*^ → ℝ^*m*^, is a non-linear mapping determined by the physical structure and parameters of the used manipulator. For a redundant manipulator, *m* < *n*; this means that when *r*(*t*) is given and known, infinite corresponding θ(*t*) may exist. Moreover, due to the non-linear property of redundant manipulator, directly solving Equation (1) is extremely difficult. On the contrast, solving Equation (1) in velocity level or acceleration level gives a simpler way. For the velocity level, Equation (1) can be transformed into

(2) $$\begin{array}{l} \dot{r}\left(t\right)=J\left(\theta \left(t\right)\right)\overset{∙}{\theta }\left(t\right), \end{array}$$

where *J*(θ(*t*)) ∈ ℝ^*m* × *n*^ is Jacobian matrix. *ṙ*(*t*) and $\overset{∙}{\theta }\left(t\right)$ correspond to the derivatives of *r*(*t*) and θ(*t*), respectively, denoting Cartesian and joint velocity, respectively.

Computing the derivatives of Equation (2), the acceleration level kinematics is described as

(3) $$\begin{array}{l} \ddot{r}\left(t\right)=J\left(\theta \left(t\right)\right)\ddot{\theta }\left(t\right)+\overset{∙}{J}\left(\theta \left(t\right)\right)\overset{∙}{\theta }\left(t\right), \end{array}$$

where $\overset{∙}{J}\left(\theta \left(t\right)\right)$ is a time derivative of *J*(θ(*t*)). $\ddot{r}\left(t\right)$ and $\ddot{\theta }\left(t\right)$ are the derivatives of *ṙ*(*t*) and $\overset{∙}{\theta }\left(t\right)$, respectively, denoting acceleration of the manipulator in Cartesian and joint space, respectively. For simplicity, in the following sections, $\overset{∙}{J}\left(\theta \left(t\right)\right)$, *J*(θ(*t*)), *r*(*t*), *ṙ*(*t*), $\ddot{r}\left(t\right)$, θ(*t*), $\overset{∙}{\theta }\left(t\right)$, and $\ddot{\theta }\left(t\right)$ are abbreviated to $\overset{∙}{J}$, *J*, *r*, *ṙ*, $\ddot{r}$, θ, $\overset{∙}{\theta }$, and $\ddot{\theta }$, respectively.

### 2.2. Obstacle Avoidance

#### 2.2.1. Basic Description

Based on the bound box theory, the robot manipulator and obstacle can be simplified as the mathematical geometry. For example, the plane manipulator can be abstracted as a combination of cylinders, and the obstacles are abstracted as spheres, cylinders, cuboids, or a combination of them (Yue et al., 2015). By describing the manipulator and obstacle as point sets, the distance between them is transformed into the point-to-point distance. Assume that *A* and *B* are Cartesian coordinates of one of the points on a manipulator and an obstacle, respectively, given a safety distance *d*; in principle, if ||*AB*|| ≥ *d* is always satisfied during robot movement, the safety (collision-free) between the robot and obstacle will be ensured, where $||AB||=\sqrt{{\left(A-B\right)}^{T}\left(A-B\right)}$ denotes the Euclidean norm.

**Figure 1:**
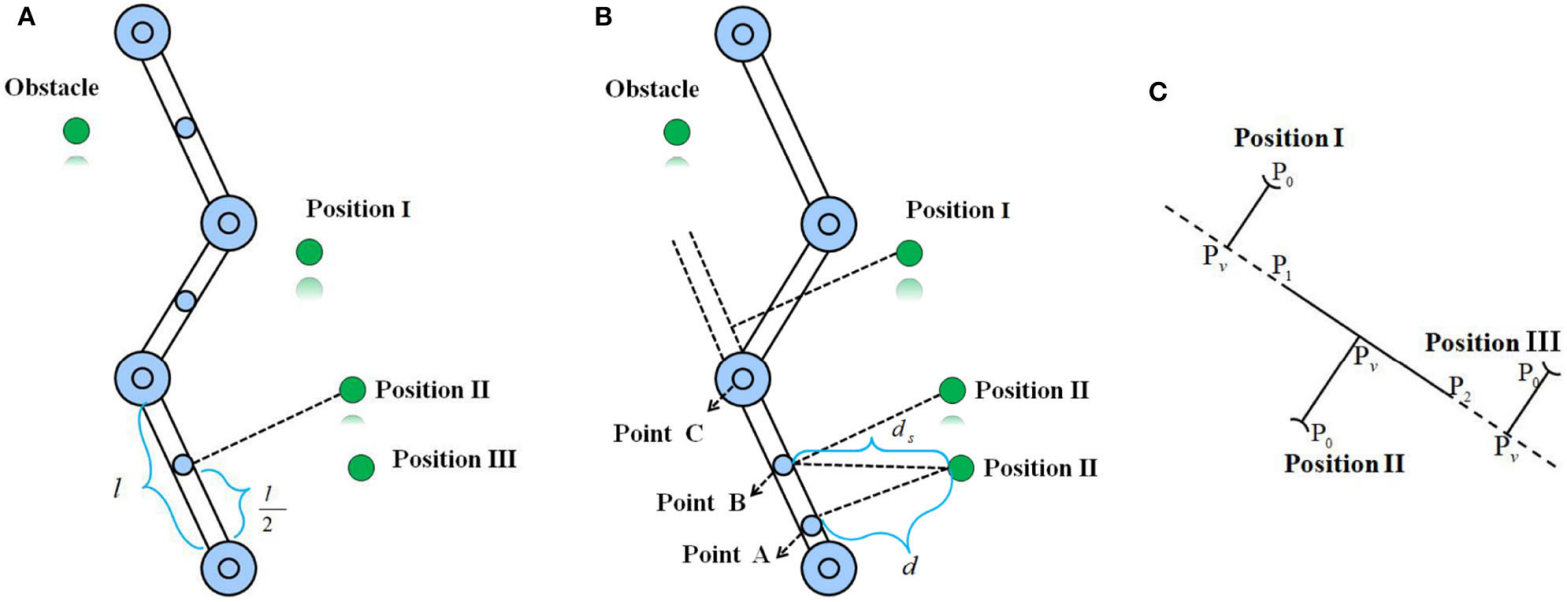
The basic idea of two obstacle avoidance schemes where the green denotes the obstacle, and the blue denotes the critical point selected from a link of a manipulator. **(A)** Uniform point selection. **(B)** The proposed nearest point selection method in this paper. **(C)** Three possible situations corresponding to the nearest point collision avoidance strategy.

As the DH parameters of a manipulator are given, Cartesian coordinates of the critical points located in manipulator joint centers are easier to compute. By setting certain criteria, critical points on links of a manipulator can also be obtained. However, how to select the representative points on both the manipulator and obstacles is challenging. Selecting abundant points will increase the computational costs and is not necessary. A method is to uniformly choose critical points on a manipulator (as shown in Figure 1A), which is introduced in Xu et al. (2019b) and Zhou et al. (2019). The basic idea is that the critical point is chosen in the center of a link of a manipulator, based on the joint angle information and link length, and Cartesian coordinates of the critical point can be computed. Although we reduced the computational complexity, we found that this way caused a possibility that the chosen points did not collide with the obstacle; in practice, the collision had already happened due to the distance from the chosen points to the obstacle being greater than the shortest distance between them.

As shown in Figure 1B, when the obstacle is located in Position III based on the uniform point selection, B will be adopted to determine whether the collision with the obstacle occurred. If the safety distance is just set as *d* + *a* and *d* < *d* + *a* < *d*_*s*_, where *a* is a positive constant, for B, the collision-free will be determined. However, for point A, the manipulator will collide with the obstacle. Therefore, motivated by it, in this study, we aim to find the nearest points on every link of the manipulator to the obstacle to ensure the collision-free. Utilizing the vector relationship between geometric elements, the method is simple and easy to implement. Assuming that *P*_1_ and *P*_2_ are the coordinates of two critical points in the center of two joints connecting a link of the manipulator, *P*_0_ are Cartesian coordinates of the detected critical point from the encountered obstacle (as shown in Figure 1C where the manipulator link and the obstacle are simplified as a segment and a point, respectively). Assume that *P*_*v*_ is a projection of *P*_0_ to segment *P*_1_*P*_2_. If $\lambda =\vec{P_{1}P_{v}}/\vec{P_{1}P_{2}}\in \left[0,1\right]$ (that is to say, *P*_0_ is located in Position II), where $\vec{\cdot }$ denotes the directional vector, then the nearest point is *P*_*v*_ with the minimal distance *d*_min_ = ||*P*_0_*P*_*v*_||. Otherwise, *d*_min_ = {||*P*_0_*P*_1_||, ||*P*_0_*P*_2_||}_min_. For λ < 0, the nearest point is *P*_2_ (i.e, *P*_0_ is located in Position III), for λ > 1, the nearest point *P*_1_ will be returned.

*Remark*1: Note that, in this paper, Cartesian coordinates of the critical points on the obstacle are known by default. In real life, the real-time measurement of the surrounding obstacles is easy to achieve by use of a camera, and the related achievements have been reported in Carloni et al. (2013) and Zhang et al. (2015).

#### 2.2.2. Inequality Formalization on an Acceleration Level

Assume that *A* is the nearest point on a link of a manipulator to the obstacle and *B* denotes the mass center of the obstacle. To ensure safety between them, the inequality ||*AB*|| ≥ *d* is required to hold. For this purpose, define *e* = ||*AB*|| − *d*, and an inequality in velocity level is constructed as follows:

(4) $$\begin{array}{l} \frac{d||AB||}{dt}\geq -k_{1}e, \end{array}$$

where *k*_1_ is a positive constant that is used to scale the convergence rate of the error. Due to $\overset{∙}{A}=J\overset{∙}{\theta }$ and

(5) $$\begin{array}{l} \frac{d||AB||}{dt}=\frac{d}{dt}\sqrt{{\left(A-B\right)}^{T}\left(A-B\right)}={\vec{\left||BA|\right|}}^{T}\left(\overset{∙}{A}-\overset{∙}{B}\right), \end{array}$$

where $\vec{\left||BA|\right|}={\left(A-B\right)}^{T}/||A-B||\in {\mathbb{R}}^{1\times m}$ is the unit vector of $\vec{A-B}$, *Ȧ* is the velocity of point *A* in joint space, and *J* ∈ ℝ^*m* × *n*^ is the Jacobian matrix of *A*; we can obtain

(6) $$\begin{array}{l} \begin{array}{c} {\vec{\left||BA|\right|}}^{T}\left(\overset{∙}{A}-\overset{∙}{B}\right)\geq -k_{1}e,\\ {\vec{\left||BA|\right|}}^{T}\left(J\overset{∙}{\theta }-\overset{∙}{B}\right)\geq -k_{1}e,\\ {\vec{\left||BA|\right|}}^{T}\left(J\overset{∙}{\theta }\right)\geq -k_{1}e+{\vec{\left||BA|\right|}}^{T}\overset{∙}{B}, \end{array} \end{array}$$

let $-{\vec{\left||BA|\right|}}^{T}J=J_{o}\in {\mathbb{R}}^{1\times n}$, $k_{1}e-{\vec{\left||BA|\right|}}^{T}\overset{∙}{B}=C$, Equation (6) can be summarized as

(7) $$\begin{array}{l} J_{o}\overset{∙}{\theta }\leq C. \end{array}$$

The velocity-level collision avoidance inequality, i.e., Equation (7), is obtained, and it has been proven to have the ability to avoid collision between the static and dynamic obstacles in Zhou et al. (2019) and Xu et al. (2019b). Much like the velocity level, by constructing

(8) $$\begin{array}{l} \frac{d}{dt}\left(\frac{d||AB||}{dt}+k_{1}e\right)\geq -k_{2}\left(\frac{d||AB||}{dt}+k_{1}e\right), \end{array}$$

then,

(9) $$\begin{array}{l} \begin{array}{c} \frac{d}{dt}\left(\frac{d||AB||}{dt}+k_{1}e\right)\geq -k_{2}\left(\frac{d||AB||}{dt}+k_{1}e\right)\\ \frac{d}{dt}\left({\vec{\left||BA|\right|}}^{T}\left(J\overset{∙}{\theta }-\overset{∙}{B}\right)+k_{1}e\right)\geq -k_{2}\left({\vec{\left||BA|\right|}}^{T}\left(J\overset{∙}{\theta }-\overset{∙}{B}\right)+k_{1}e\right)\\ -J_{o}\ddot{\theta }-{\overset{∙}{J}}_{o}\overset{∙}{\theta }-{\vec{\left||BA|\right|}}^{T}\ddot{B}+k_{1}\left(-J_{o}\overset{∙}{\theta }-{\vec{\left||BA|\right|}}^{T}\overset{∙}{B}\right)\geq \\ -k_{2}\left(-J_{o}\overset{∙}{\theta }-{\vec{\left||BA|\right|}}^{T}\overset{∙}{B}+k_{1}e\right)\\ J_{o}\ddot{\theta }+{\overset{∙}{J}}_{o}\overset{∙}{\theta }+{\vec{\left||BA|\right|}}^{T}\ddot{B}+k_{1}\left(J_{o}\overset{∙}{\theta }+{\vec{\left||BA|\right|}}^{T}\overset{∙}{B}\right)\leq \\ -k_{2}\left(J_{o}\overset{∙}{\theta }+{\vec{\left||BA|\right|}}^{T}\overset{∙}{B}-k_{1}e\right), \end{array} \end{array}$$

therefore, we can obtain the obstacle avoidance inequality in acceleration level:

(10) $$\begin{array}{l} \begin{array}{c} J_{o}\ddot{\theta }\leq -k_{2}\left(J_{o}\overset{∙}{\theta }+{\vec{\left||BA|\right|}}^{T}\overset{∙}{B}-k_{1}e\right)\\ \;-k_{1}\left(J_{o}\overset{∙}{\theta }+{\vec{\left||BA|\right|}}^{T}\overset{∙}{B}\right)-\overset{∙}{J_{o}}\overset{∙}{\theta }-{\vec{\left||BA|\right|}}^{T}\ddot{B}. \end{array} \end{array}$$

where $-{\vec{\left||BA|\right|}}^{T}J=J_{o}$. Let the right side of inequality (10) be denoted by μ; Equation (10) is then equivalent to

(11) $$\begin{array}{l} J_{o}\ddot{\theta }\leq \mu . \end{array}$$

So far, the construction of the inequality collision avoidance strategy on the acceleration level, i.e., Equation (11), is completed.

### 2.3. QP Problem Statement

For a redundant manipulator, due to the redundancy, it is possible to perform the primary and secondary tasks simultaneously. In view of *m* < *n*, many solutions satisfying Equation (11) exist. To choose a better solution from them, the secondary task can be set as the optimization of some performance indices such as joint velocity minimization, joint acceleration minimization, joint jerk minimization, etc. In this study, the acceleration level kinematic control of the redundant manipulator was considered, and the joint-acceleration-norm minimization was thus chosen. On one hand, in terms of practical industrial applications, the robot is often expected to perform some repetitive tasks such as palletizing and welding. To make the kinematic control of manipulator repetitive, the RMP scheme was proposed and investigated in Zhang et al. (2009), Xiao and Zhang (2013), and Jin et al. (2018), and it was constructed as the minimization of the displacements between the θ(*t*) and θ(0), where θ(0) denotes the initial joint angle. On the other hand, to avoid the joint drift problem, another performance index, i.e., the repetitive motion planning, was also adopted in this paper:

1. Minimum acceleration norm (MAN):
  (12) $$\begin{array}{l} U={\ddot{\theta }}^{T}\ddot{\theta }/2. \end{array}$$
2. Repetitive motion planning (RMP):
  (13) $$\begin{array}{l} U={\left(\ddot{\theta }+d_{1}\left(\theta -\theta \left(0\right)\right)\right)}^{T}\left(\ddot{\theta }+d_{1}\left(\theta -\theta \left(0\right)\right)\right)/2, \end{array}$$

where *d*_1_ > 0 is designed as a positive constant determined by the designer based on the experimental results, which is used to scale the magnitude of the displacements θ − θ(0). Parameters θ and θ(0) denote the current joint angle and the initial joint angle of the manipulator, respectively.

Let η = *d*_1_(θ − θ(0)), assigning a weight ω_1_ = 0.5 and ω_2_ = 0.5 to the MAN and the RMP schemes, respectively; ω_1_ + ω_2_ = 1, the bi-criteria acceleration level obstacle avoidance, and kinematic control problem of a redundant manipulator are formulated as an QP problem as follows:

(14a) $$\begin{array}{ll} \text{min} & \;{\ddot{\theta }}^{T}\ddot{\theta }/4+{\left(\ddot{\theta }+\eta \right)}^{T}\left(\ddot{\theta }+\eta \right)/4, \end{array}$$

(14b) $$\begin{array}{ll} \text{s.t.} & \;J\ddot{\theta }={\ddot{r}}_{d}-\overset{∙}{J}\overset{∙}{\theta }, \end{array}$$

(14c) $$\begin{array}{l} J_{o}\ddot{\theta }\leq \mu , \end{array}$$

(14d) $$\begin{array}{l} \theta^{-}\leq \theta \leq \theta^{+}, \end{array}$$

(14e) $$\begin{array}{l} {\overset{∙}{\theta }}^{-}\leq \overset{∙}{\theta }\leq {\overset{∙}{\theta }}^{+}, \end{array}$$

(14f) $$\begin{array}{l} {\ddot{\theta }}^{-}\leq \ddot{\theta }\leq {\ddot{\theta }}^{+}, \end{array}$$

where Equation (14a) denotes the objective function to be minimized. Equations (14b) and (14c) denote the motion planning scheme and obstacle avoidance scheme, respectively. Equations (14d)–(14f) are the physical constraints. Parameters $\ddot{\theta }$, $\overset{∙}{\theta }$, θ denote joint acceleration vector, joint velocity vector and joint angle vector of the robot manipulator, respectively. θ^−^, ${\overset{∙}{\theta }}^{-}$, ${\ddot{\theta }}^{-}$ and θ^+^, ${\overset{∙}{\theta }}^{+}$, ${\ddot{\theta }}^{+}$ are lower bound and upper bound of θ, $\overset{∙}{\theta }$, $\ddot{\theta }$, respectively. *r*_*d*_ are the desired trajectory that the robot is expected to track. ${\ddot{r}}_{d}$ is the time derivation of *ṙ*_*d*_, and *ṙ*_*d*_ is the derivation of *r*_*d*_.

## 3. QP Reformulation and RNN Controller

### 3.1. QP Reformulation

For Equation (14b), to achieve a higher tracking accuracy to the desired trajectory, a feedback is introduced, and Equation (14b) is rewritten as

(15) $$\begin{array}{l} J\ddot{\theta }={\ddot{r}}_{d}-\overset{∙}{J}\overset{∙}{\theta }-\beta \left(J\overset{∙}{\theta }-{\overset{∙}{r}}_{d}\right)-\gamma \left(r-r_{d}\right), \end{array}$$

where β > 0 ∈ ℝ and γ > 0 ∈ ℝ are the feedback gains, and *r* is the actual trajectory achieved by manipulator under the designed controller. In addition, For Equations (14d)–(14f), it is obvious that they are located at different levels, which makes it impossible to directly solve Equation (14). Following Guo and Zhang (2014, 2019), Equations (14d)–(14f) can be incorporated in the acceleration level, i.e.,

(16) $$\begin{array}{l} \begin{array}{c} \xi^{+}=\min\left\{\kappa_{1}\left(\theta^{+}-\vartheta -\theta \right),\kappa_{2}\left({\overset{∙}{\theta }}^{+}-\overset{∙}{\theta }\right),{\ddot{\theta }}^{+}\right\},\\ \xi^{-}=\max\left\{\kappa_{1}\left(\theta^{-}+\vartheta -\theta \right),\kappa_{2}\left({\overset{∙}{\theta }}^{-}-\overset{∙}{\theta }\right),{\ddot{\theta }}^{-}\right\}, \end{array} \end{array}$$

where ϑ > 0 ∈ ℝ, κ_1_ > 0 ∈ ℝ, and κ_2_ > 0 ∈ ℝ. The QP problem (14) can thus be reformulated:

(17a) $$\begin{array}{ll} \text{min} & \;{\ddot{\theta }}^{T}\ddot{\theta }/4+{\left(\ddot{\theta }+\eta \right)}^{T}\left(\ddot{\theta }+\eta \right)/4, \end{array}$$

(17b) $$\begin{array}{ll} \text{s.t.} & \;J\ddot{\theta }={\ddot{r}}_{d}-\overset{∙}{J}\overset{∙}{\theta }-\beta \left(J\overset{∙}{\theta }-{\overset{∙}{r}}_{d}\right)-\gamma \left(r-r_{d}\right), \end{array}$$

(17c) $$\begin{array}{l} J_{o}\ddot{\theta }\leq \mu , \end{array}$$

(17d) $$\begin{array}{l} \xi^{+}=\min\left\{\kappa_{1}\left(\theta^{+}-\vartheta -\theta \right),\kappa_{2}\left({\overset{∙}{\theta }}^{+}-\overset{∙}{\theta }\right),{\ddot{\theta }}^{+}\right\}, \end{array}$$

(17e) $$\begin{array}{l} \xi^{-}=\max\left\{\kappa_{1}\left(\theta^{-}+\vartheta -\theta \right),\kappa_{2}\left({\overset{∙}{\theta }}^{-}-\overset{∙}{\theta }\right),{\ddot{\theta }}^{-}\right\}. \end{array}$$

*Remark*2: The weight factors of both the MAN and RMP schemes are set at the same value, meaning that the MAN and RMP schemes are viewed as equally important. For different weights ω_1_ and ω_2_ = 1 − ω_1_, the minimized objective function is different.

### 3.2. RNN Controller

In this part, we would design an RNN-based dynamic controller to solve Equation (17) recursively. Specifically, for Equation (17), a lagrange function is defined as

(18) $$\begin{array}{l} L={\ddot{\theta }}^{T}\ddot{\theta }/4+{\left(\ddot{\theta }+\eta \right)}^{T}\left(\ddot{\theta }+\eta \right)/4+\lambda_{1}^{T}\left(B_{right}-J\ddot{\theta }\right)+\lambda_{2}^{T}\left(J_{o}\ddot{\theta }-\mu \right), \end{array}$$

where $B_{right}={\ddot{r}}_{d}-\overset{∙}{J}\overset{∙}{\theta }-\beta \left(J\overset{∙}{\theta }-{\overset{∙}{r}}_{d}\right)-\gamma \left(r-r_{d}\right)$, λ_1_ and λ_2_ is the Lagrange multiplier. Based on the KKT conditions, the optimal solution of Equation (18) can be equivalently rewritten as

(19a) $$\begin{array}{l} \ddot{\theta }=P_{\Omega }\left(\ddot{\theta }-\frac{\partial L}{\partial \ddot{\theta }}\right), \end{array}$$

(19b) $$\begin{array}{l} J\ddot{\theta }=B_{right}, \end{array}$$

(19c) $$\begin{array}{l} \{\begin{array}{c} \lambda_{2}=0\;\text{if }J_{o}\ddot{\theta }\leq \mu ,\;\\ \lambda_{2}>0\;\text{othewise}.\; \end{array} \end{array}$$

where *P*_Ω_ is a projection operation to a set Ω, and *P*_Ω_(*x*) = argmin_*y* ∈ Ω_||*y* − *x*|| Li et al. (2016). Equation (19c) can be further written as

(20) $$\begin{array}{l} \lambda_{2}=\max\left(\left(\lambda_{2}+J_{o}\ddot{\theta }-\mu \right),0\right). \end{array}$$

**Figure 2:**
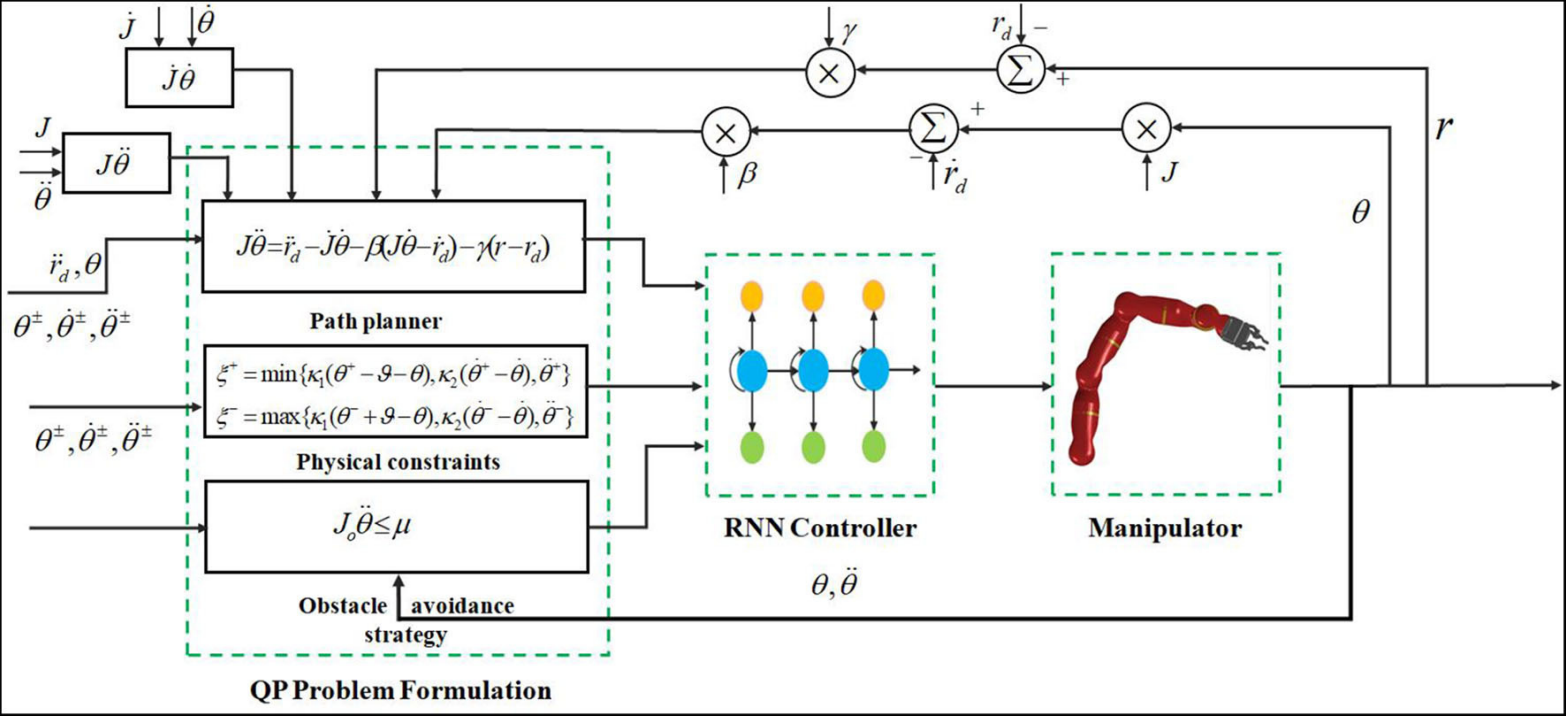
Block diagram of the acceleration-level kinematic motion control of redundant manipulator with obstacle avoidance (17c) and physical constraints (17d)-(17e) under the designed RNN controller (21).

The designed RNN controller is:

(21a) $$\begin{array}{l} \epsilon \overset{.}{\theta }=-\ddot{\theta }+P_{\Omega }\left(-\frac{1}{2}\eta +J^{T}\lambda_{1}-J_{o}^{T}\lambda_{2}\right), \end{array}$$

(21b) $$\begin{array}{l} \epsilon {\overset{∙}{\lambda }}_{1}=J\ddot{\theta }-B_{right}, \end{array}$$

(21c) $$\begin{array}{l} \epsilon {\overset{∙}{\lambda }}_{2}=\max\left(\left(J_{o}\ddot{\theta }-\mu +\lambda_{2}\right),0\right)-\lambda_{2}, \end{array}$$

where ϵ > 0 is a constant that is used to scale the convergence rate of the neural network. Figure 2 shows a block diagram of the acceleration-level kinematic motion control of a redundant manipulator with obstacle avoidance (17c) and physical constraints (17d)-(17e) under the designed RNN controller (21).

## 4. Simulation

In this paper, the simulation experiment was performed on a plane four-DOF robot manipulator to validate the feasibility of the control scheme Equation (21). Table 1 gives the corresponding DH parameters of the employed manipulator and the parameter values involved in the simulative experiment, respectively.

**Table 1:** The D-H parameter of the robot manipulator employed in this paper and simulation parameters setup.

**Link**	**a(m)**	**α(rad)**	**d(m)**	**Parameter**	**Value**	**Parameters**	**Value**
1	0.296	0	0	*k*_1_	7	*k*_2_	7
2	0.296	0	0	ϵ	0.002	ϑ	0.1
3	0.296	0	0	κ_1_	20	κ_2_	20
4	0.212	0	0	β	20	γ	20
				θ^−^	-2(rad)	θ^+^	2(rad)
				${\overset{∙}{\theta }}^{-}$	-2(rad/s)	${\overset{∙}{\theta }}^{+}$	2(rad/s)
				${\ddot{\theta }}^{-}$	-2(rad/s)	${\ddot{\theta }}^{+}$	2(rad/s^2^)
				*d*	0.1(m)	*d*_1_	10

*The left side is the DH parameter. The right side is simulation parameters involved in the simulative experiments*.

### 4.1. Circle Trajectory Tracking

#### 4.1.1. Static Obstacle

**Figure 3:**
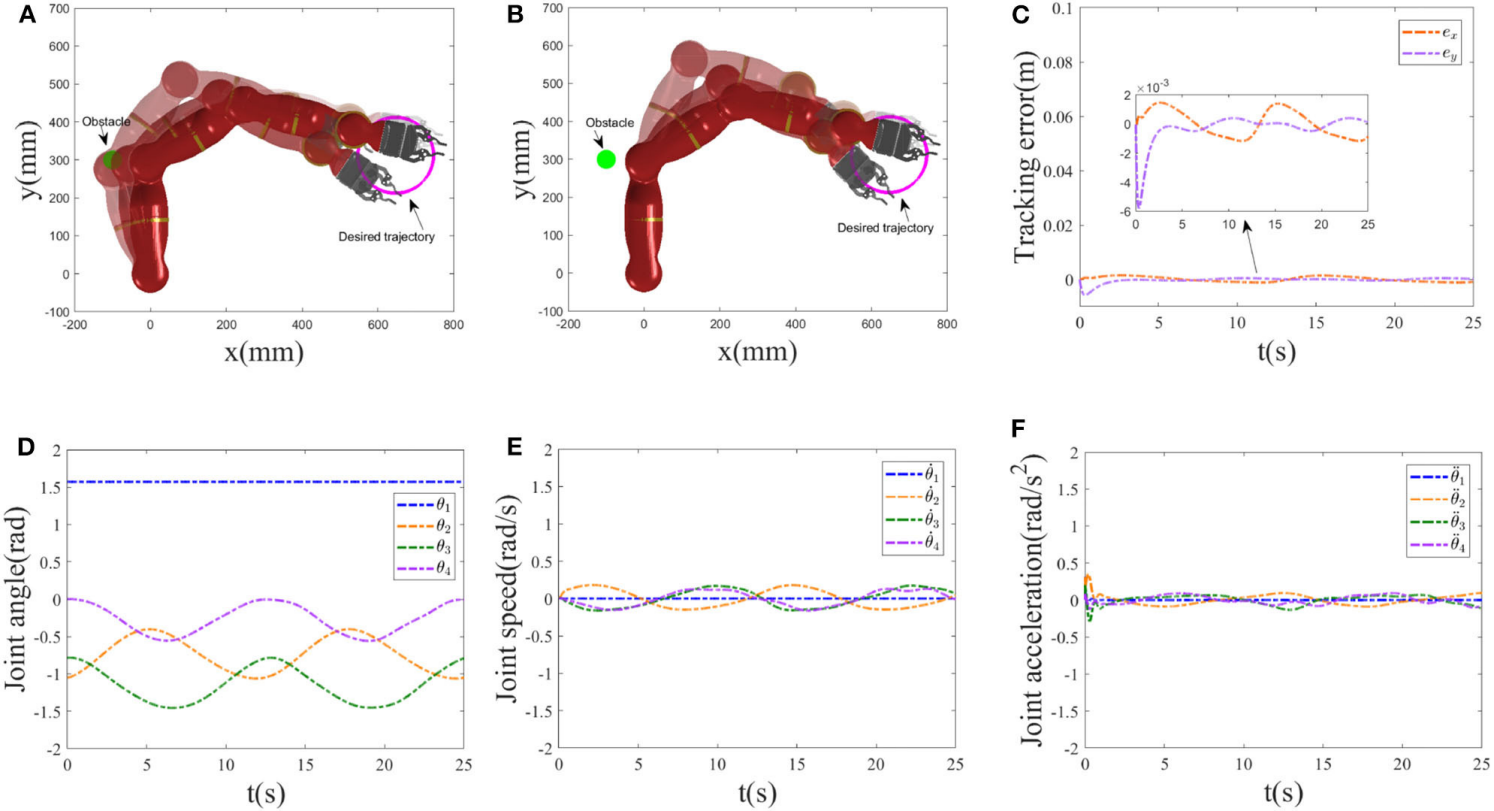
Static obstacle collision avoidance results. **(A)** Trajectory tracked by the manipulator without Equation (17c) at *t* = 1, 6, 9 and *t* = 13*s*. **(B–F)** Simulation results achieved by the manipulator with Equation (17c). **(B)** Tracked trajectory. **(C)** Tracking error at *x*-axis, *y*-axis. **(D)** Joint angle profiles. **(E)** Joint velocity profiles. **(F)** Joint acceleration profiles.

In this experiment, the robot is expected to track a circle trajectory with definition of $r_{d}={\left[0.6470+0.1\cos\left(0.5t\right),0.3125+0.1\sin\left(0.5t\right)\right]}^{T}$ whose radius is 0.1. Assume that position of the obstacle is centered at [−0.1, 0.3]^*T*^m. The initial joint angle is chosen as *q*(0) = [π/2, −π/3, −π/4, 0]^*T*^rad with the joint velocity and joint acceleration initializing as zero. The simulation time is set as 25*s* with step size being 0.001. Collision avoidance and trajectory tracking results are shown in Figure 3, where the single static obstacle is considered. Figures 3A,B show the trajectory tracked by the manipulator under RNN controller without and with the obstacle avoidance strategy Equation (17c), respectively, where the corresponding tracking results are used at *t* = 1, 6, 9 and *t* = 13*s*. Following them, when not considering the collision avoidance, though the manipulator successfully tracks the desired circle trajectory, distances between the obstacle and both the first and second links of the manipulator are small, and this allows the collision between them to happen. For practical industrial applications, this control method will inevitably lead to the tracking failure of the expected behavior. After introducing the obstacle avoidance strategy, as the distance between the nearest point on the manipulator and the encountered environmental obstacle, and as the obstacle is less than the setting safety distance 0.1, the inequality Equation (17c) comes in the control command, enabling the manipulator to escape the obstacle (see Figure 3B); under the path-tracking controller Equation (17b), the robot moves along the desired trajectory as expected with a promising tracking error being the 10^−3^ order (see Figure 3C). As the initial point of the end-effector of the manipulator coincides with the expected tracking trajectory, the tracking error is always satisfying from the start to the end of simulation. Figures 3D–F show the joint angles, joint velocities, and joint accelerations profiles, respectively. Among them, the lines are relatively smooth and not sharp, and they do not exceed the setting the bound constrains.

#### 4.1.2. Verification of RMP

**Figure 4:**
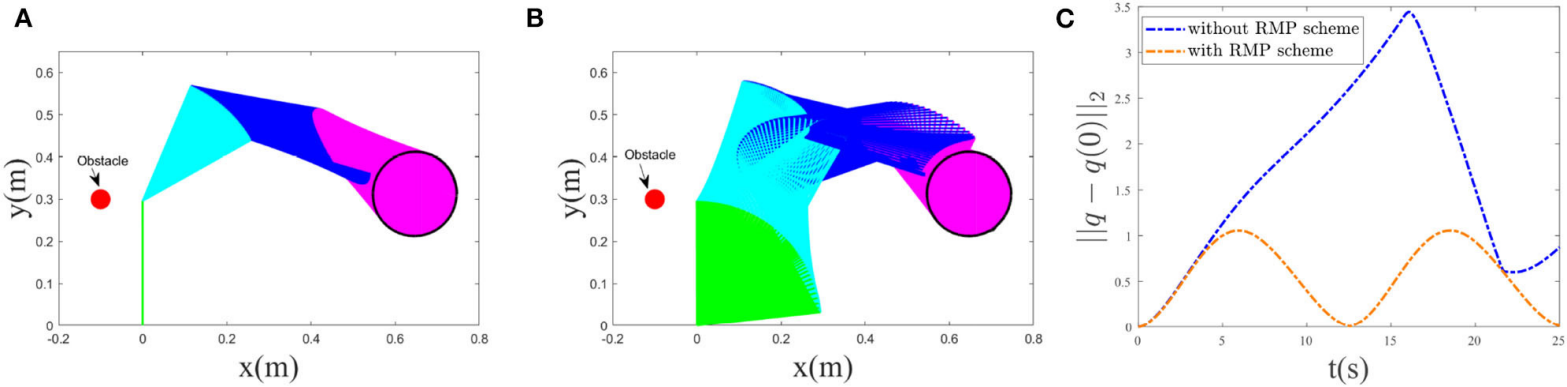
Comparative results between bi-criteria scheme considering the MAN and RMP and the MAN scheme. **(A)**: Tracking trajectory result corresponding to bi-criteria scheme; **(B)** Tracking result corresponding to MAN scheme. **(C)**: Comparison of ||*q* − *q*(0)||_2_ with and without RMP scheme.

Now, we start to validate the effectiveness of the RMP scheme. In this paper, the performance index was chosen as a bi-criteria optimization, i.e., a weighted combination of the MAN scheme Equation (12) and the RMP scheme Equation (13). The desired trajectory tracking result corresponding to the bi-criteria scheme is shown in Figure 4A, and the one corresponding to the MAN scheme is illustrated in Figure 4B. Comparing Figures 4A,B, the joint-drift problem at the acceleration level can be seen to be solved by considering the RMP scheme. In addition, a comparison between ||*q* − *q*(0)||_2_ with and without the RMP scheme is illustrated in Figure 4C, showing that, for the scheme considering RMP, ||*q* − *q*(0)||_2_ would be guaranteed to converge to zero when *t* = *T*, 2*T* and change periodically. If not considering the RMP scheme, ||*q* − *q*(0)||_2_ increases when *t* = *T*, 2*T* and is haphazard. Moreover, based on the simulative results shown in Figures 3D,E, we observe that when *t* = *T*, 2*T*, joint angles and joint velocities of the manipulator are guaranteed to return to their initial configurations. The RMP scheme can therefore be said to be effective.

#### 4.1.3. Dynamic Obstacle

**Figure 5:**
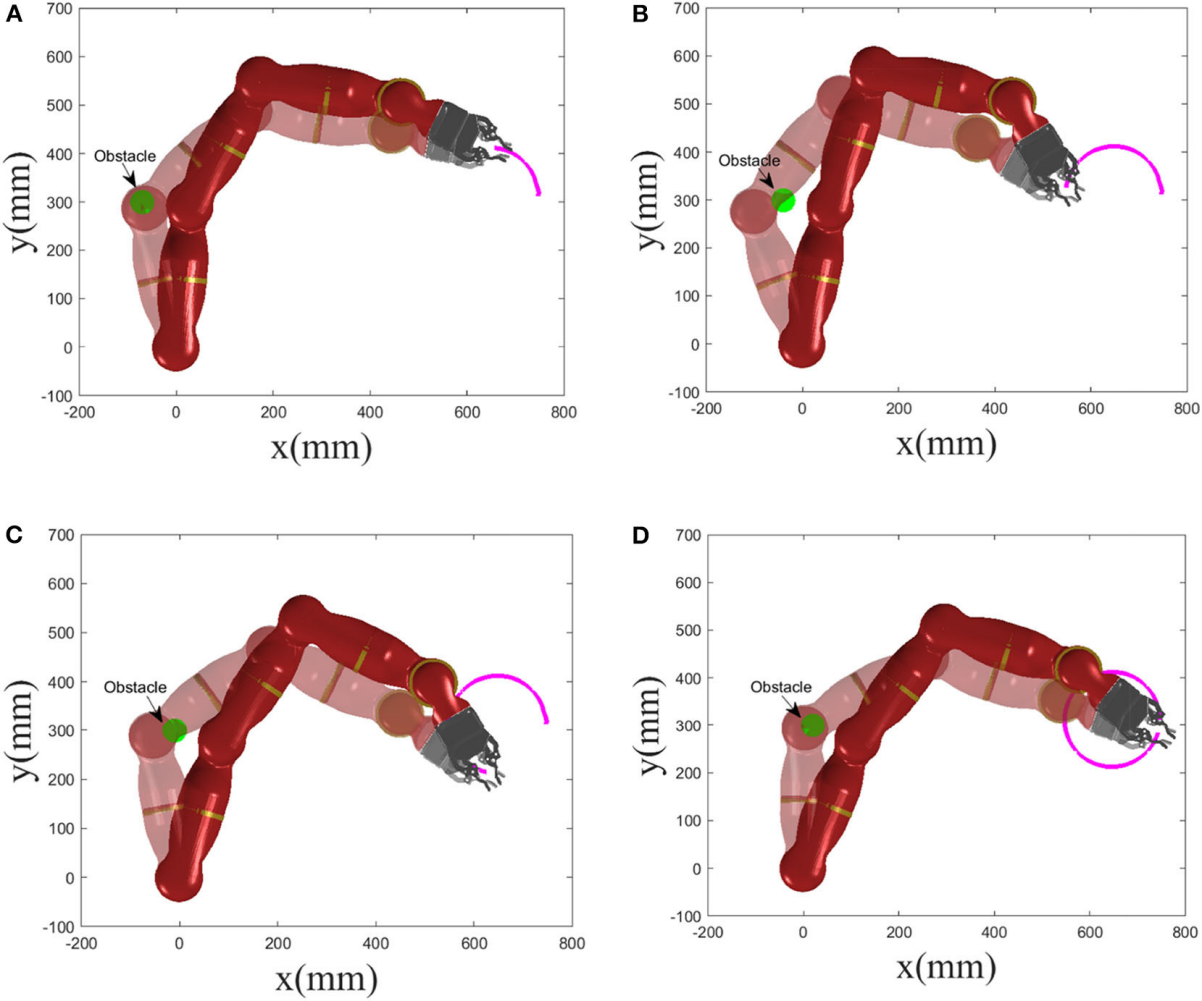
Snapshots of manipulator avoiding a dynamic obstacle at different time *t*, where the real shadow denotes collision avoidance result with Equation (17c), and the virtual shadow corresponds to ones without Equation (17c). **(A)** *t* = 3 s. **(B)** *t* = 6 s. **(C)** *t* = 9 s. **(D)** *t* = 12 s.

**Figure 6:**
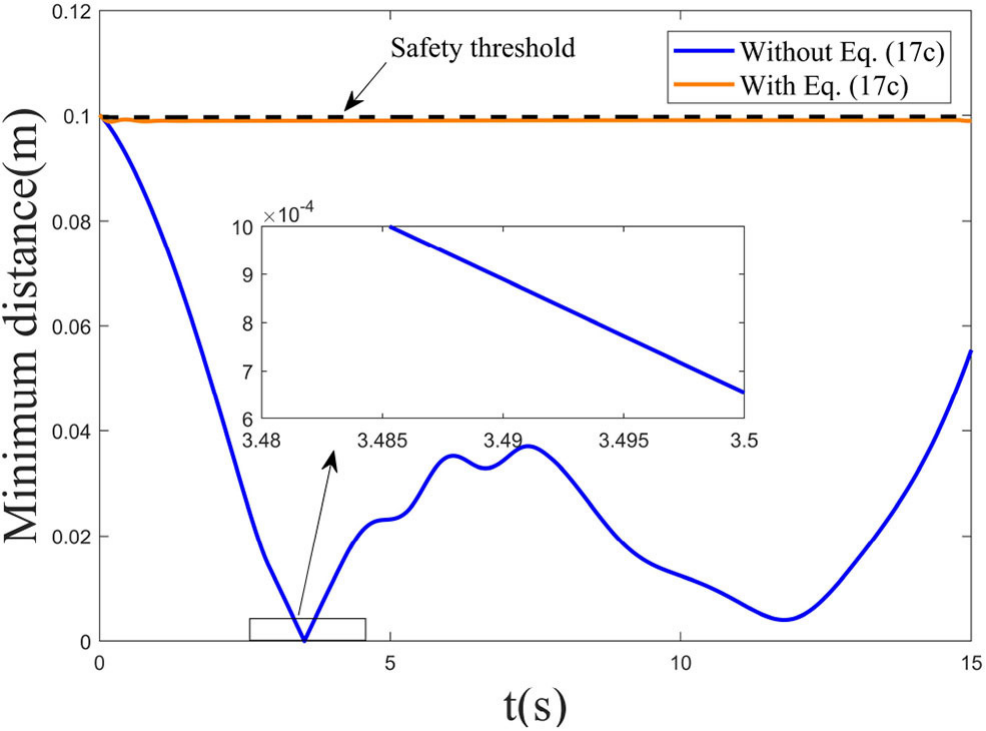
Minimum distance profile between the robot and the considered dynamic obstacle achieved by the controller without and with Equation (17c).

Pedestrians or other objects with dynamic property may break into the motion range of the robot. In this part, we consider the collision avoidance between the robot and a dynamic obstacle, and snapshots of the manipulator avoiding a dynamic obstacle at different time *t* are given in Figure 5, where the real shadow denotes the collision avoidance result achieved by the manipulator under the RNN dynamic controller with the inequality collision avoidance strategy Equation (17c), and the virtual shadow corresponds to ones without Equation (17c). The motion trajectory of the considered dynamic obstacle is set as [−0.1 + 0.01*t*, 0.3]^*T*^ with simulation time being 15*s*. Macroscopically, when *t* = 3*s*, 6*s*, 9*s* and 12*s*, if not considering the collision avoidance, the manipulator collides with the dynamic obstacle. After introducing the collision avoidance strategy, under the control of the controller, the robot escapes the obstacle by changing its joint angles and being maintained outside the non-safety distance. To further show the effectiveness of the collision avoidance scheme Equation (17c), Table 2 gives the corresponding Cartesian coordinates of the nearest point on the manipulator to the obstacle obtained by the controller with and without Equation (17c) at different time *t* and the distance between the nearest point and the obstacle. Obviously, without Equation (17c), the distance is significantly less than the safety distance 0.1, meaning that the collision will happen with high probability. By contrast, after introducing Equation (17c), the collision avoidance scheme comes in the control command, and enables the manipulator to escape the obstacle and maintain a safe distance. Based on Table 2, under the control of the dynamic controller, the distances between the nearest point on the manipulator and the obstacle maintain 0.099, which is very close to 0.1. In addition, we give the minimum distance profile between the manipulator and the dynamic obstacle achieved by the controller without and with Equation (17c) for illustration, as shown in Figure 6. It is more obvious and intuitive than Table 2, if not taking the collision avoidance strategy Equation (17c) into account, and the robot would collide with the obstacle at *t* = 3.5*s* owing to the distance being 7 × 10^−4^m. It is therefore concluded that the proposed collision avoidance strategy is effective.

**Table 2:** A dynamic obstacle is considered: the nearest point on the manipulator to the obstacle obtained by the controller with and without the obstacle avoidance scheme Equation (17c) at different time *t* and the distance between the nearest point and the obstacle.

**Time (s)**	**Nearest point with Equation (17c)**	**Distance (m)**	**Nearest point without Equation (17c)**	**Distance (m)**
*t* = 1	[0.0089, 0.2959]^*T*^	0.0990	[−0.0107, 0.2958]^*T*^	0.0794
*t* = 3	[0.0285, 0.2903]^*T*^	0.0990	[−0.0616, 0.2910]^*T*^	0.0123
*t* = 6	[0.0570, 0.2803]^*T*^	0.0990	[−0.0677, 0.3215]^*T*^	0.0351
*t* = 9	[0.0845, 0.2704]^*T*^	0.0991	[−0.0210, 0.3151]^*T*^	0.0187
*t* = 12	[0.1112, 0.2612]^*T*^	0.0991	[0.0178, 0.3039]^*T*^	0.0045
*t* = 15	[0.1450, 0.2722]^*T*^	0.0989	[0.0136, 0.3417]^*T*^	0.0554

*Remark 3:* Compared to the setting safety threshold 0.1m, the distances between the nearest point to the obstacle on the manipulator and the obstacle at different time were maintained at 0.099 in the dynamic obstacle avoidance experiment. We observed that the minimal distance achieved by the controller was a somewhat smaller than 0.1, and this is attributed to the sampling interval adopted in the simulation experiment. Compared to the generated results without Equation (17c), the proposed collision avoidance strategy can help the robot to avoid collision with the obstacle on the whole, and the slight difference can thus be ignored.

### 4.2. Three-Ring Trajectory Tracking

**Figure 7:**
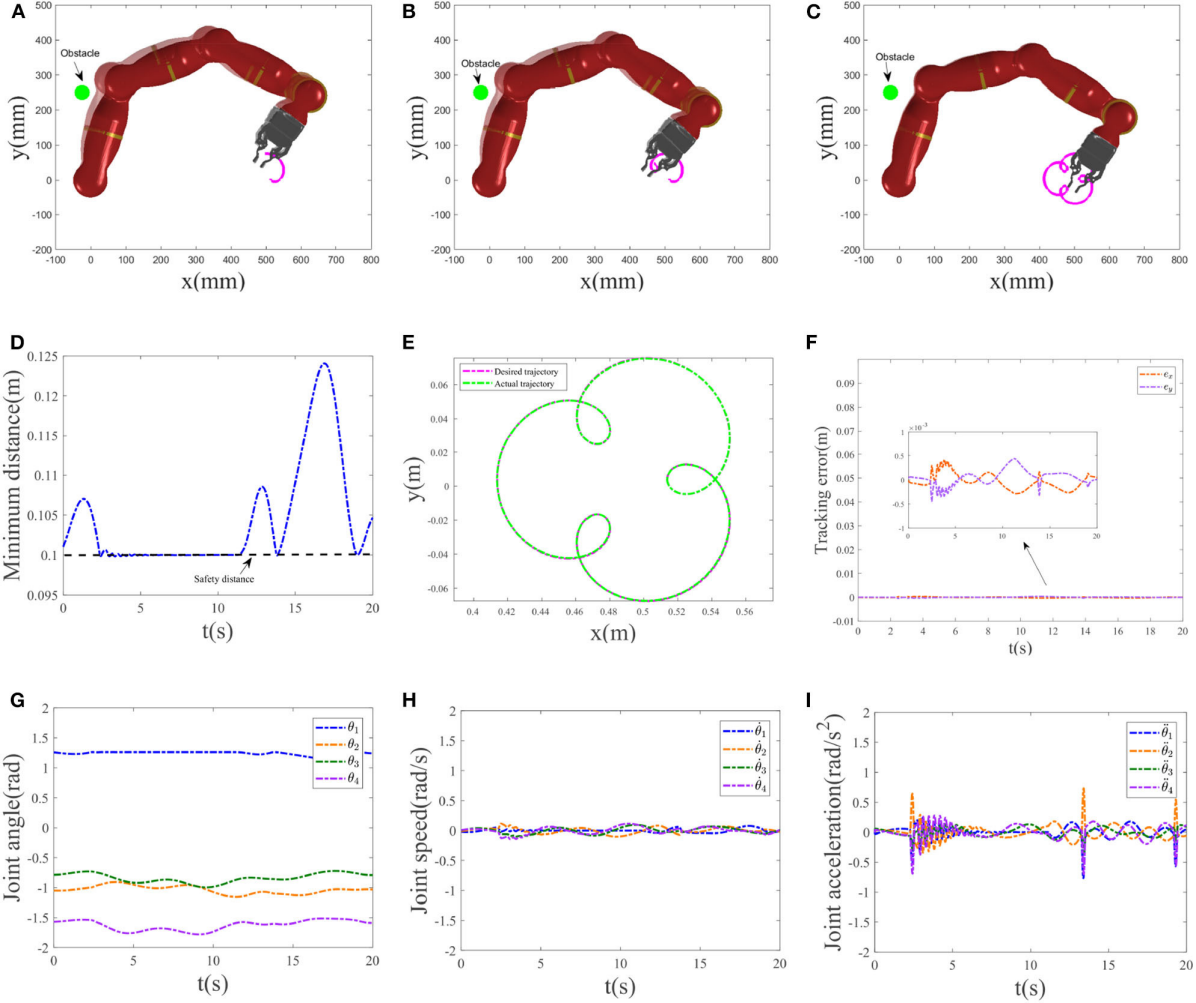
Obstacle collision avoidance results. **(A–C)** Snapshots of the manipulator avoiding a static obstacle at *t* = 4*s*, *t* = 8*s*, and *t* = 20*s*, respectively. **(D)** Minimum distance profile. **(E)** Trajectory tracked by the manipulator and the desired trajectory. **(F)** Tracking error at *x*-axis, *y*-axis. **(G)** Joint-angle profiles. **(H)** Joint-velocity profiles. **(I)** Joint-acceleration profiles.

To further validate the feasibility of the dynamic controller [Equation (17)] integrating path tracking and obstacle avoidance strategies, in this experiment, the robot tracks a three-ring trajectory. The tracked trajectory is defined as $r_{d}={\left[0.05\cos\left(\pi t/10\right)-0.025\cos\left(4\pi t/10\right)+0.4888,0.05\sin\left(\pi t/10\right)-0.025\sin\left(4\pi t/10\right)+0.0040\right]}^{T}$. Position of the obstacle is located on [−0.025, 0.25]^*T*^m. The initial joint angle is valued as *q*(0) = [π/2.5, −π/3, −π/4, −π/2]^*T*^rad, and the initial joint velocity is set as [−0.0210, −0.0101, 0.0032, 0.0092]^*T*^rad/s. Other experimental parameters are the same as the previous circle trajectory tracking. The simulation time is set as 20*s* with step size being 0.001. Static obstacle collision avoidance and trajectory tracking results are illustrated in Figure 7. Figures 7A–C show snapshots of manipulator avoiding a static obstacle at different time *t* = 4*s*, *t* = 8*s*, and *t* = 20*s*, respectively, where the real shadow denotes collision avoidance result with Equation (17c), the virtual shadow corresponds to ones without Equation (17c). We can observe that when not considering the collision avoidance scheme Equation (17c), the distance between the first link of the manipulator and the obstacle is tiny (e.g., when for *t* = 8, the minimal distance is 0.0785 with the nearest point being [0.0516, 0.2330]^*T*^). After introducing Equation (17c), compared to the previous, the distance between them is enlarged and maintained outside the non-safety region (see Figure 7D). Figure 7D gives the distance between the nearest point on the first link of the manipulator to the obstacle and the obstacle. When *t* = 4*s* and *t* = 8*s*, the distance between them is maintained as 0.1, i.e., the setting safety distance. For *t* = 8, the neatest point returned by the computer is [0.0702, 0.2195]^*T*^. Except when successfully avoiding the obstacle, the robot also accomplishes the desired three-ring path tracking as expected. Based on Figures 7E,F, it is obvious that the actual trajectory achieved by the manipulator is coincident with the desired trajectory, and the tracking errors at *x*-axis and *y*-axis reach 10^−3^ level. Figures 7G,I show the joint-angle profiles, joint-velocity profiles, and joint-acceleration profiles of the manipulator, respectively. Following them, therefore, we can say that the proposed obstacle avoidance scheme Equation (17c) and the designed RNN dynamic controller Equation (17) are effective for solving the kinematic motion problem of a redundant manipulator at the acceleration level.

**Figure 8:**
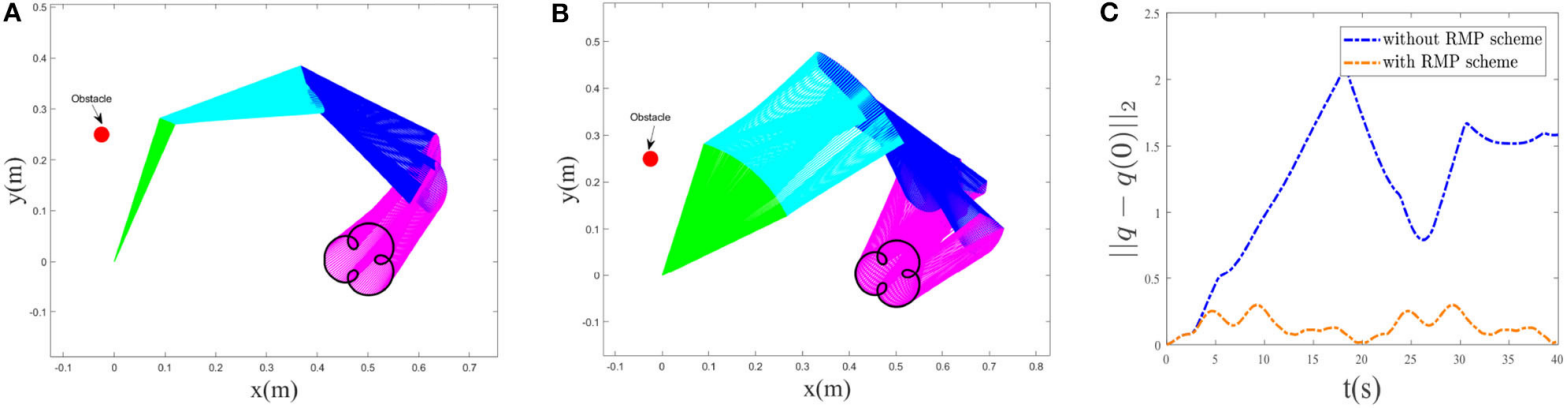
Comparative results between bi-criteria scheme considering the MAN and RMP, and the MAN scheme. **(A)** Tracking trajectory result corresponding to bi-criteria scheme; **(B)** Tracking result corresponding to MAN scheme. **(C)** Comparison of ||*q* − *q*(0)||_2_ with and without RMP scheme.

Comparative results between the bi-criteria scheme and the MAN scheme are shown in Figure 8. As the movement period is 20*s*, in this experiment, the simulation time is set as 40*s*. In addition, *d*_1_ = 15. Figures 8A,B show the tracking trajectory result corresponding to bi-criteria scheme and MAN scheme, respectively. A comparison between ||*q* − *q*(0)||_2_ with and without the RMP scheme is illustrated in Figure 8C. For the scheme considering RMP, ||*q* − *q*(0)||_2_ would be guaranteed to converge to zero when *t* = *T*, 2*T* and changes periodically. If is not considering the RMP scheme, when *t* = *T*, 2*T*, the current joint-angle state *q* does not return the initial joint-angle state *q*(0).

**Figure 9:**
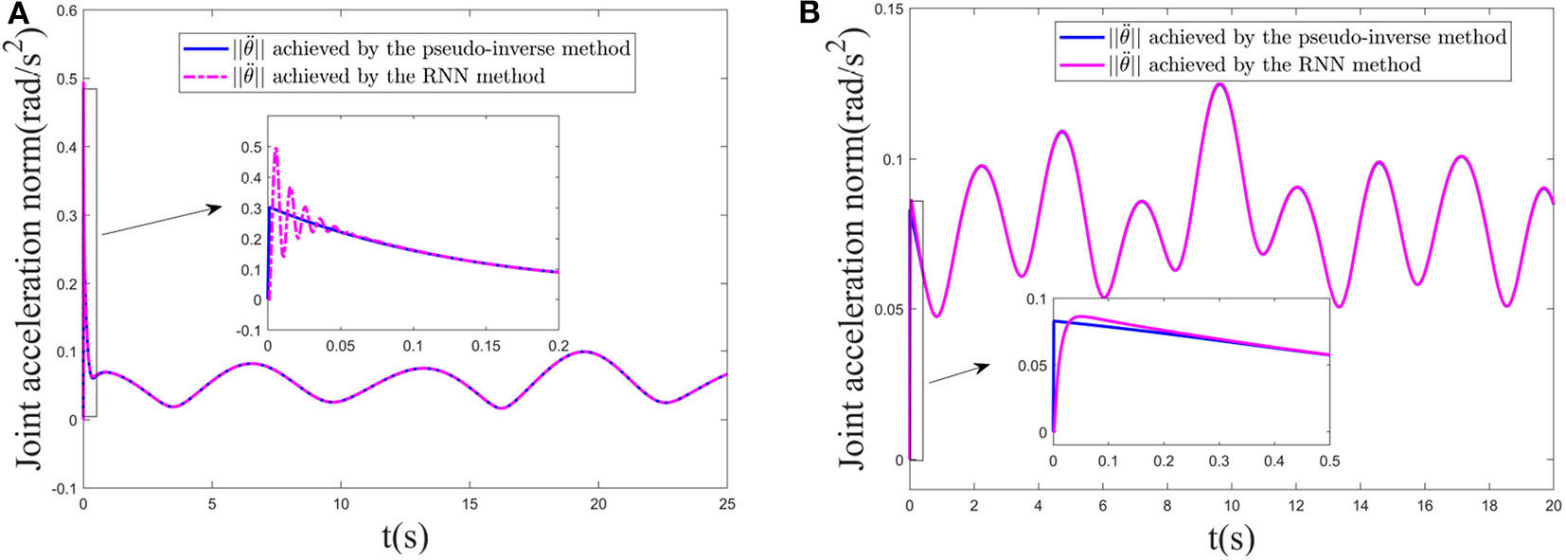
Joint acceleration norm $\left||\ddot{\theta }|\right|$ comparison achieved by the pseudo-inverse method and the RNN-based method proposed in this paper, respectively. **(A)** Circle trajectory tracking. **(B)** Three-ring trajectory tracking.

*Remark 4:* As one of the important performance indices, here, we start to show the effectiveness of the MAN scheme. Figure 9 gives comparative results of the joint-acceleration norm $\left||\ddot{\theta }|\right|$ achieved by the pseudo-inverse method and the RNN-based method proposed in this paper for two trajectories. Figure 9A corresponds to the circle trajectory tracking experiment. Figure 9B corresponds to the three-ring trajectory tracking experiment. Note that, in this experiment, the cost function only considers the MAN scheme to show the effectiveness of the MAN scheme, not involving the RMP and the collision avoidance. In general, the pseudo-inverse method is deemed as a persuasive solution, therefore, it is employed to compare with our RNN-based method. Following Figure 9A, it is observed that $\left||\ddot{\theta }|\right|$ quickly coincides with one achieved by the pseudo-inverse method although it is slightly different at initial time. The conclusion is also same for the three-ring trajectory (as shown in Figure 9B), consequently, the effectiveness of the MAN scheme is validated. In addition, the pseudo-inverse method does not handle the physical constraints such as joint angles, joint accelerations, consequently, the RNN-based method in this paper is adopted.

### 4.3. Comparison

As described in the previous sections, obstacle avoidance of the redundant manipulator has been investigated for decades, and the research has been fruitful. However, the existing products mainly focus on the velocity level. At present, only a small amount of attention is paid to the obstacle avoidance of the redundant manipulator at the acceleration level (not to mention the bi-criteria acceleration-level obstacle avoidance). There are few related works that have been reported. In this study based on the QP optimization, we investigated the bi-criteria acceleration-level obstacle avoidance of the redundant manipulator. To highlight the proposed controller scheme in this paper, comparisons between our scheme and the existing QP-based acceleration level obstacle avoidance schemes were conducted, and the comparative results are illustrated in Table 3. In Xiao and Zhang (2013), the obstacle avoidance scheme was not considered. For their works proposed by Guo and Zhang (2014, 2019) and Guo and Li (2016), in their collision-avoidance schemes, the inner and outer safety thresholds were considered. In Guo et al. (2018), a noise-tolerant obstacle avoidance strategy was introduced by proposing an integration-enhanced error function. As this paper is not investigated from the perspective of QP, it is not listed in Table 3. Following Table 3, this study first investigated the bi-criteria performance optimization-based acceleration-level obstacle avoidance of the redundant manipulator from the QP perspective. Moreover, the dynamic obstacle as also considered.

**Table 3:** Comparison between the proposed scheme in this paper and the existing QP-based acceleration level obstacle avoidance schemes.

**Methods**	**Performance indices**	**Physical constraints**	**Static obstacle**	**Dynamic obstacle**
This paper	Bi-criteria	Yes	Yes	Yes
Guo and Zhang (2019)	MAN	Yes	Yes	No
Guo and Zhang (2014)	MAN	Yes	Yes	No
Xiao and Zhang (2013)	RMP	Yes	/	/
Guo and Li (2016)	MAN	No	Yes	No

*/ denotes the obstacle avoidance is not considered in Xiao and Zhang (2013)*.

Combining all simulative results, in summary, the proposed collision avoidance scheme has the ability to find the nearest point on the manipulator to obstacle, and it can enable the manipulator to avoid collision with the environmentally static and dynamic obstacles. Under the designed RNN controller, the manipulator also accurately achieves the desired trajectory tracking task.

## 5. Conclusions

We shed some light on the acceleration-level kinematic motion control problem of the redundant manipulator with obstacle avoidance in this paper. An improved inequality obstacle avoidance method is introduced, and it can find the nearest point on every link of a manipulator to an obstacle. By keeping the minimal distance between them outside the non-safety region at all times, the safety is ensured. Minimizing the combination integrating the joint-acceleration norm and repetitive motion planning as the objective function, a QP optimization problem is established where the desired motion behavior and obstacle avoidance are formulated as equality and inequality constraints rebuilt at the acceleration level. The inherent physical constraints of the manipulator are also incorporated. An RNN-based neural dynamic controller is designed to solve the resultant QP problem. Simulative results performing on four-link planer manipulator validate the feasibility of the designed control scheme, when the minimal distance between robot and obstacle violates the setting safety criticality, the collision avoidance strategy come in the control command, the robots successfully avoid collision with the environmental obstacles. If no collision is detected, the robot performs the desired trajectory tracking task with a promising tracking error. In this paper, we only considered the obstacle avoidance problem of a single redundant manipulator. For the multiple robot system, the obstacle avoidance scheme should not only consider collision between the manipulator and the environment, but also collision between the manipulators each other. This is a challenge problem. In the future work, the obstacle avoidance problem of multiple robot manipulators system will be considered.

## Data Availability Statement

The original contributions presented in the study are included in the article/supplementary material, further inquiries can be directed to the corresponding author/s.

## Author Contributions

WZ developed the idea for this study, derived mathematical equations, and wrote to the paper. Some drawings in this paper and the program code were completed by XL. XC and XS completed the corresponding MATLAB simulation. GT contributed to refining the paper and proposed amendments. All authors contributed to the article and approved the submitted version.

## Conflict of Interest

WZ, XC, and XS are employed by the company Foshan Longshen Robotics LTD. The remaining authors declare that the research was conducted in the absence of any commercial or financial relationships that could be construed as a potential conflict of interest.
